# Combined Blockade of ADP Receptors and PI3-Kinase p110β Fully Prevents Platelet and Leukocyte Activation during Hypothermic Extracorporeal Circulation

**DOI:** 10.1371/journal.pone.0038455

**Published:** 2012-06-06

**Authors:** Stefanie Krajewski, Julia Kurz, Tobias Geisler, Karlheinz Peter, Hans Peter Wendel, Andreas Straub

**Affiliations:** 1 Department of Anesthesiology and Intensive Care Medicine, University of Tübingen, Tübingen, Germany; 2 Department of Cardiology, University of Tübingen, Tübingen, Germany; 3 Atherothrombosis and Vascular Biology, Baker IDI Heart and Diabetes Institute, Melbourne, Victoria, Australia; 4 Department of Thoracic, Cardiac and Vascular Surgery, University of Tübingen, Tübingen, Germany; Heart Center Munich, Germany

## Abstract

Extracorporeal circulation (ECC) and hypothermia are used to maintain stable circulatory parameters and improve the ischemia tolerance of patients in cardiac surgery. However, ECC and hypothermia induce activation mechanisms in platelets and leukocytes, which are mediated by the platelet agonist ADP and the phosphoinositide-3-kinase (PI3K) p110β. Under clinical conditions these processes are associated with life-threatening complications including thromboembolism and inflammation. This study analyzes effects of ADP receptor P_2_Y_12_ and P_2_Y_1_ blockade and PI3K p110β inhibition on platelets and granulocytes during hypothermic ECC. Human blood was treated with the P_2_Y_12_ antagonist 2-MeSAMP, the P_2_Y_1_ antagonist MRS2179, the PI3K p110β inhibitor TGX-221, combinations thereof, or PBS and propylene glycol (controls). Under static *in vitro* conditions a concentration-dependent effect regarding the inhibition of ADP-induced platelet activation was found using 2-MeSAMP or TGX-221. Further inhibition of ADP-mediated effects was achieved with MRS2179. Next, blood was circulated in an *ex vivo* ECC model at 28°C for 30 minutes and various platelet and granulocyte markers were investigated using flow cytometry, ELISA and platelet count analysis. GPIIb/IIIa activation induced by hypothermic ECC was inhibited using TGX-221 alone or in combination with P_2_Y blockers (p<0.05), while no effect of hypothermic ECC or antiplatelet agents on GPIIb/IIIa and GPIbα expression and von Willebrand factor binding was observed. Sole P_2_Y and PI3K blockade or a combination thereof inhibited P-selectin expression on platelets and platelet-derived microparticles during hypothermic ECC (p<0.05). P_2_Y blockade alone or combined with TGX-221 prevented ECC-induced platelet-granulocyte aggregate formation (p<0.05). Platelet adhesion to the ECC surface, platelet loss and Mac-1 expression on granulocytes were inhibited by combined P_2_Y and PI3K blockade (p<0.05). Combined blockade of P_2_Y_12_, P_2_Y_1_ and PI3K p110β completely inhibits hypothermic ECC-induced activation processes. This novel finding warrants further studies and the development of suitable pharmacological agents to decrease ECC- and hypothermia-associated complications in clinical applications.

## Introduction

Under physiological conditions, platelets play a fundamental role in hemostasis, prevention of blood loss, and healing of vascular injury. However, dysfunctional platelets can cause serious problems like abnormal thrombus formation and consecutive vessel occlusion as well as severe bleeding complications, which are all feared side effects of extracorporeal circulation (ECC) [1], [2].

ECC is employed in many cardiac surgical procedures to ensure gas exchange and to maintain stable circulatory parameters of the patient. In addition, hypothermia ranging between 28°C and 32°C is routinely employed during cardiac operations in addition to ECC to increase the ischemia tolerance of the patient. Shear stress, contact of blood with the artificial surfaces of the ECC circuit as well as hypothermia are all known to be associated with platelet activation, which results in disturbed platelet function and associated complications [1], [3], [4]. Furthermore, activated platelets can trigger an inflammatory response through interactions with leukocytes [5]. These platelet-leukocyte interactions are mainly mediated by binding of the platelet surface receptor P-selectin to its counter receptor P-selectin glycoprotein ligand-1 (PSGL-1) on leukocytes. Subsequently, upregulation and activation of the Mac-1 receptor (CD11b/CD18) on leukocytes is induced as a result of the P-selectin-PSGL-1 interaction [5], [6]. Furthermore, it has been shown that CD40 ligand, which is shed from platelets upon activation, also promotes Mac-1 upregulation [7].

Inhibition of platelet activation is a possible approach to inhibit platelet dysfunction and related detrimental effects during ECC. One pharmacological strategy to inhibit platelet activation is blockade of the platelet ADP receptors P_2_Y_12_ and P_2_Y_1_ [8], [9].

We have recently shown that ADP plays a major role in ECC- and hypothermia-induced platelet activation [10]. Inhibition of platelet granule release could be achieved during hypothermic ECC via P_2_Y_12_ blockade [11]. Nevertheless, despite effective platelet protection by P_2_Y_12_ blockade, still higher degrees of platelet activation compared to baseline values were observed. Furthermore, platelet adhesion to the ECC surface and therefore platelet loss could not be prevented. Consequently, in addition to ADP other factors obviously activate platelets during ECC. In this regard, shear-induced activation of platelets is another important factor during ECC [4], [12]. Shear triggers a signaling pathway, which includes activation of the class Ia phosphoinositide-3-kinase (PI3Ks) p110β isoform. This results in activation of the platelet fibrinogen receptor GPIIb/IIIa and platelet aggregate formation [13], [14], [15], [16].

On the basis of these data, we hypothesize that substantial platelet protection during ECC and hypothermia may be achieved by combined inhibition of P_2_Y_12_, P_2_Y_1_ and PI3K p110β. To prove this, we first defined effective doses of the P_2_Y_12_ antagonist 2-MeSAMP and the PI3K p110β blocker TGX-221 to achieve substantial inhibition of platelet activation *in vitro*. Afterwards, the effect of single and combined blockade of ADP receptors and PI3K p110β on platelet activation, platelet adhesion to the ECC surface, their interaction with granulocytes as well as subsequent granulocyte activation was investigated in human whole blood employing an *ex vivo* ECC model at hypothermia (28°C).

## Results

### Concentration-dependent Inhibition of ADP-induced P-selectin Expression using 2-MeSAMP and TGX-221 and the Effect of MRS2179

**Figure 1:**
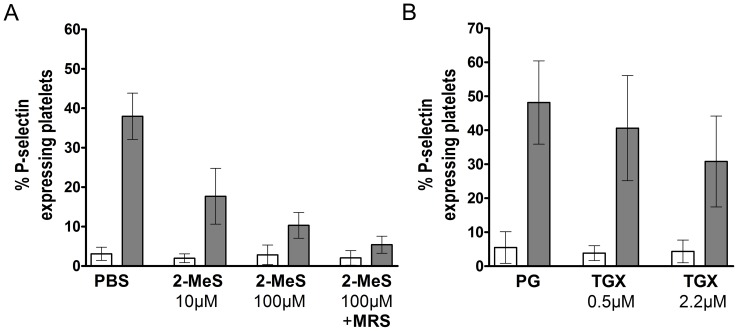
Concentration-dependent inhibition of ADP-induced P-selectin expression using 2-MeSAMP and TGX-221. Heparinized human whole blood (n = 4) was treated for 5 minutes at 37°C with (A) PBS as control, 2-MeSAMP (10 and 100 µM; “2-MeS”) or a combination (100 µM each) of 2-MeSAMP and MRS2179 and (B) propylene glycol (“PG”) as control or TGX-221 (0.5 or 2.2 µM; “TGX”). Afterwards platelets were activated using ADP (final concentration: 20 µM) and the percentage of P-selectin expressing platelets under a pre-set histogram marker was analyzed in flow cytometry using an anti-P-selectin mAb. Data are given as means and SD.

Treatment of whole blood with different concentrations of 2-MeSAMP (10 and 100 µM) showed that ADP-induced (final ADP concentration: 20 µM) P-selectin expression is more potently inhibited with higher antagonist concentrations (Figure 1A). The addition of MRS2179 (100 µM) in the 2-MeSAMP-treated group further decreased the expression of platelet P-selectin expression upon ADP activation (Figure 1A).

Furthermore, a TGX-221 concentration of 2.2 µM is more potently inhibiting ADP-induced P-selectin expression in human whole blood, when compared to a TGX-221 concentration of 0.5 µM (Figure 1B).

### PI3K p110β Inhibition Alone or in Combination with ADP Receptor Blockade has no Effect on Expression Levels of GPIIb/IIIa and GPIbα as Well as on vWF Binding of Platelets, but Reduces GPIIb/IIIa Activation during Hypothermic ECC

**Figure 2:**
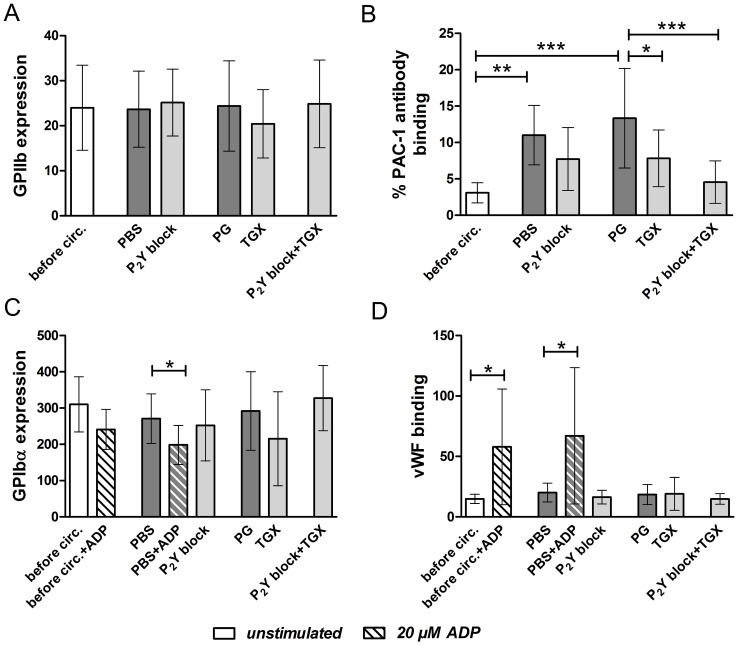
GPIIb/IIIa and GPIbα expression as well as vWF binding are not influenced by *ex vivo* hypothermic ECC and antiplatelet agents, while platelet PI3K p110β inhibition alone or in combination with P_2_Y_12_/P_2_Y_1_ receptor blockade reduce GPIIb/IIIa activation induced by hypothermic ECC. Human blood was left untreated (“before circ.”) or treated *ex vivo* with PBS (control), a combination of 2-MeSAMP and MRS2179 to block P_2_Y receptors (100 µM each; “P_2_Y block”), propylene glycol (“PG”, control), TGX-221 to inhibit PI3K p110β (2.2 µM; “TGX”) or a combination of 2-MeSAMP, MRS2179 (100 µM each) and TGX-221 (2.2 µM; “P_2_Y block + TGX”). All treated samples were circulated in an ECC model for 30 minutes at 28°C. Expression of GPIIb (A; n = 4), activated GPIIb/IIIa (B; n = 6), GPIbα (C; n = 6) as well as vWF binding (D; n = 4) were evaluated in flow cytometry using specific antibodies. ADP stimulation (20 µM) before and after hypothermic ECC was performed as positive control for GPIbα expression (C) and vWF binding (D) in the PBS group. Geometric mean fluorescence values of fluorescently labeled antibodies are given in diagrams as means and SD; not normally distributed data were analyzed using a non-parametrical test (Friedman test with Dunǹs multiple comparison test; A, D); normally distributed data (B, C) were compared using RM-ANOVA with Bonferroni’s multiple comparison test; *p<0.05; **p<0.01; ***p<0.001.

Platelet activation is accompanied by a conformational change of the platelet GPIIb/IIIa receptor into a high-affinity conformation, which enables binding of ligands including fibrinogen and vWF [19]. Using specific antibodies we analyzed (1) the general expression of GPIIb/IIIa on the platelet surface and (2) the GPIIb/IIIa activation state. Basic GPIIb/IIIa expression was neither influenced by hypothermic ECC nor by antiplatelet agents (Figure 2A), while hypothermic ECC induced a significant increase in GPIIb/IIIa activation in controls (p<0.01; Figure 2B). This effect was inhibited by PI3K p110β inhibition using TGX-221 alone (p<0.05) and more profoundly by PI3K p110β inhibition combined with P_2_Y blockade (2-MeSAMP and MRS2179; p<0.001).

The GPIbα receptor is involved in mediating primary platelet adhesion via binding to its ligand vWF [19]. Upon platelet activation GPIbα surface expression is decreased due to downregulation [20] or receptor shedding [21], [22]. Regarding the expression of the GPIbα receptor on platelets as well as vWF binding to platelets in our experiments, no effect of either hypothermic ECC or P_2_Y and PI3K p110β inhibition was observed (Figure 2C and D). It has previously been described that the platelet agonist ADP decreases GPIbα surface expression on platelets [20], [23], [24]. This finding was confirmed in our experiments and indicated intact platelet reactivity before and after hypothermic ECC in the PBS group (Figure 2C).

In addition, vWF binding was investigated on ADP-activated platelets before and after hypothermic ECC in the PBS group. ADP induced a significant increase in vWF binding at both timepoints (Figure 2D). Regarding the fact that GPIbα surface expression was downregulated at the same time, this finding is most likely due to binding of vWF to GPIIb/IIIa. In this regard, it has previously been described that platelets have other binding sites for vWF then GPIbα [25] and that vWF is a ligand of the GPIIb/IIIa receptor [19], [26].

### Hypothermic ECC-induced P-selectin Expression on Platelets and Platelet Microparticles is Profoundly Inhibited by P_2_Y and PI3K p110β Inhibitors

**Figure 3:**
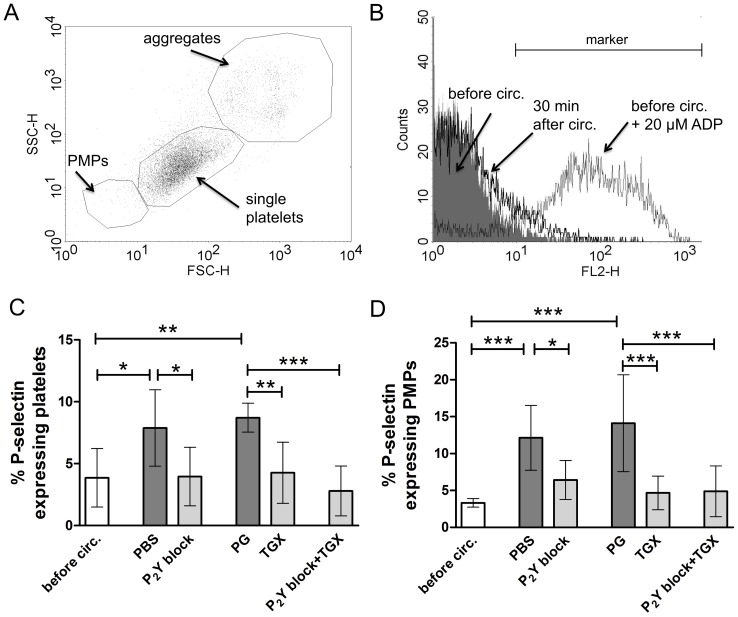
Blockade of P_2_Y_12_ and P_2_Y_1_ as well as PI3K p110β inhibition profoundly inhibits hypothermic ECC-induced P-selectin expression on platelets and platelet microparticles. Prior to (“before circ.”) and after hypothermic ECC (28°C, 30 minutes) flow cytometric analysis of P-selectin expression on platelets and PMPs was performed in groups treated with either PBS as control, 2-MeSAMP and MRS2179 (100 µM each; “P_2_Y block”), propylene glycol (“PG”) as control, TGX-221 to inhibit PI3K p110β (2.2 µM; “TGX”) or a combination of 2-MeSAMP and MRS2179 (100 µM each) as well as TGX-221 (2.2 µM; “P_2_Y block + TGX”). Representative dot plot indicating the identification of PMPs, single platelets and aggregates according to their size and granularity (A). Representative histogram overlay including a marker to identify percentages of P-selectin expressing platelets before circulation without additional stimulation (grey filled), before circulation with addition of ADP (20 µM; grey solid line) as well as 30 minutes after hypothermic ECC (black solid line) (B). Percentages of platelets (C) and PMPs (D) expressing P-selectin are depicted. Data in (C) and (D) are given as means (n = 6) and SD; groups were compared using RM-ANOVA with Bonferroni’s multiple comparison test; *p<0.05; **p<0.01, ***p<0.001.

As an indicator for α-granule release and platelet activation, P-selectin expression on the surface of platelets and platelet-derived microparticles was measured before and after hypothermic ECC. Single platelets and PMPs were identified according to their size and granularity (Figure 3A). As a positive control P-selectin expression on ADP-stimulated platelets was measured to confirm platelet reactivity before and after circulation in the control group (Figure 3B).

P-selectin expression significantly increased during hypothermic ECC in controls (p<0.05, Figure 3C). Both P_2_Y blockade and PI3K p110β inhibition alone as well as combined inhibition of ADP receptors and PI3K p110β completely inhibited this phenomenon (p<0.05).

PMPs, which express typical platelet surface receptors including P-selectin, are released from platelets upon activation [3], [27], [28]. In our experiments the total number of PMPs was not affected by hypothermic ECC or antiplatelet agents (data not shown). Nevertheless, P-selectin expression on PMPs was significantly increased by hypothermic ECC (p<0.001; Figure 3D). P_2_Y receptor blockade, PI3K p110β inhibition as well as the combination of P_2_Y receptor and PI3K p110β inhibition significantly reduced P-selectin expression on PMPs (p<0.05).

### Effects of Hypothermic ECC, P_2_Y Blockade and PI3K p110β Inhibition on Platelet-ECC Adhesion, Platelet-granulocyte Aggregate Formation and Platelet Loss

**Figure 4:**
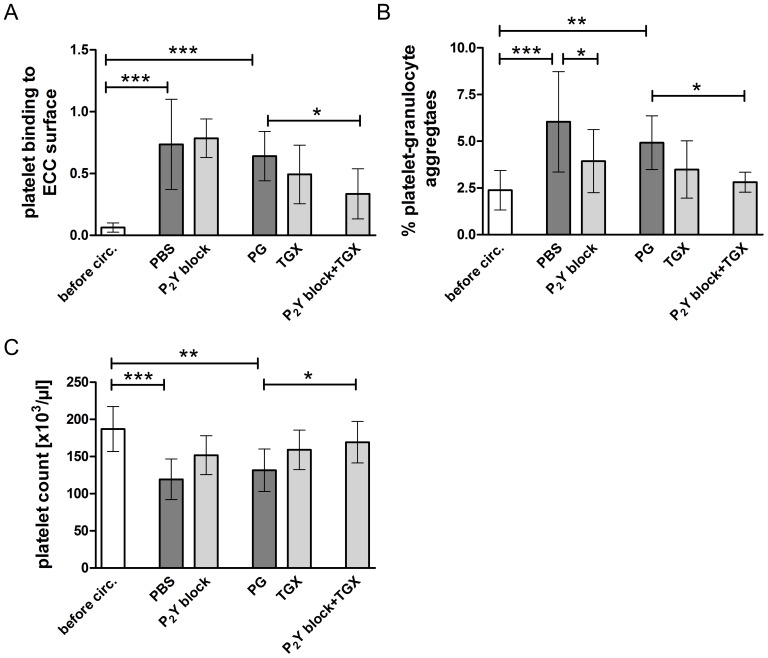
Effects of hypothermic ECC and platelet inhibitor treatment on platelet adhesion to the ECC surface, platelet-granulocyte aggregate formation and platelet counts. Human blood samples were either left untreated or treated with PBS (control group), a combination of 2-MeSAMP and MRS2179 (100 µM each; “P_2_Y block”), propylene glycol (“PG”, control group), TGX-221 (2.2 µM; “TGX”) or a combination of 2-MeSAMP, MRS2179 and TGX-221 (“P_2_Y block + TGX”). Blood, which was treated accordingly, was circulated in an *ex vivo* ECC model at 28°C for 30 minutes. A specific ELISA method was employed to detect platelet adhesion to the ECC surface (A; n = 6). Platelet-granulocyte aggregate formation was measured in flow cytometry before circulation (“before circ.”) and after circulation in all treatment groups (B; n = 4) according to the fluorescence of an anti-CD15-PE antibody on aggregates (aggregate region in figure 3A) under a pre-set histogram marker. Platelet counts were measured in all samples (C; n = 6). Data are given as means and SD; normally distributed data were compared using RM-ANOVA with Bonferroni’s multiple comparison test (A, B); not normally distributed data were analyzed using a non-parametrical test (Friedman test with Dunǹs multiple comparison test; C); *p<0.05; **p<0.01; ***p<0.001.

Hypothermic ECC induced a significant increase in platelet adhesion to the artificial ECC surface in controls (p<0.001; Figure 4A). This was only prevented by a combination of P_2_Y blockade and PI3K p110β inhibition (p<0.05), but not by P_2_Y blockade or PI3K p110β inhibition alone.

Interaction of activated platelets with leukocytes plays a pivotal role in ECC-related pro-inflammatory complications [1], [29]. We evaluated the formation of platelet-granulocyte aggregates during hypothermic ECC *ex vivo*. A significant increase in aggregate formation was found after 30 minutes of circulation at 28°C in controls (p<0.01; Figure 4B). This phenomenon was significantly inhibited by P_2_Y blockade alone (p<0.05) and by combined treatment with P_2_Y and PI3K p110β blockers (p<0.05).

A significant decrease of platelet counts was observed during hypothermic ECC (p<0.01; Figure 4C). The combination of P_2_Y blockers and PI3K p110β inhibitor significantly prevented hypothermic ECC-induced platelet loss (p<0.05).

### P_2_Y Blockade in Combination with a PI3K p110β Inhibitor Prevents Upregulation of the Mac-1 Receptor on Granulocytes

**Figure 5:**
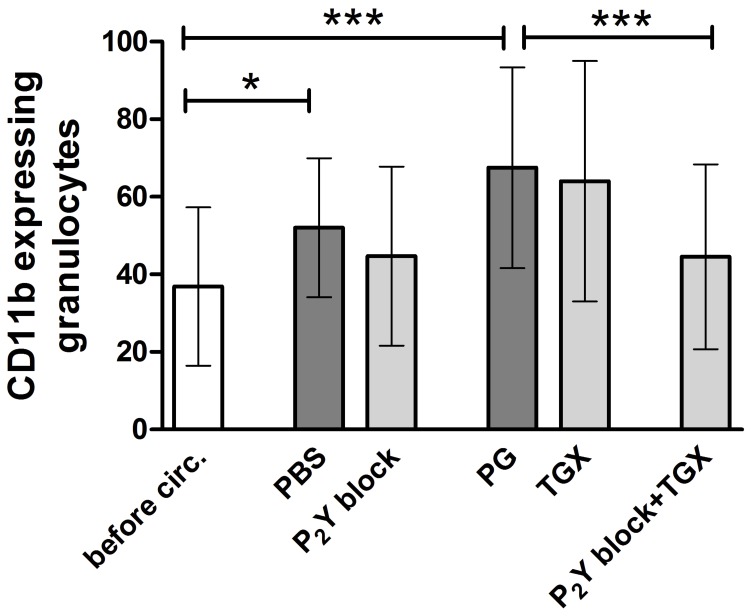
P_2_Y_12_/P_2_Y_1_ blockade in combination with TGX-221 prevents upregulation of the Mac-1 receptor on granulocytes. Granulocyte activation was measured before (“before circ.”) and 30 minutes after hypothermic ECC in groups treated with PBS (control group), 2-MeSAMP and MRS2179 (“P_2_Y block”; 100 µM each), propylene glycol (“PG”, control group), TGX-221 (“TGX”; 2.2 µM) and a combination of P_2_Y block (100 µM each) and PI3K p110β inhibition with TGX-221 (2.2 µM; “P_2_Y block + TGX”;). Mac-1 expression on granulocytes was evaluated using geometric mean values of antibody fluorescence in flow cytometry. Data are given as means (n = 6) and SD; groups were compared using RM-ANOVA with Bonferroni’s multiple comparison test; *p<0.05; ***p<0.001.

Interaction of activated platelets with leukocytes triggers the upregulation of the Mac-1 receptor on leukocytes. Mac-1 mediates leukocyte adhesion and migration as well as interaction and binding of leukocytes and platelets [30]. To evaluate granulocyte activation, we measured surface CD11b expression on granulocytes. Hypothermic ECC induced a profound increase in CD11b expression on granulocytes in controls (p<0.05; Figure 5). This was significantly decreased by P_2_Y blockade in combination with PI3K p110β inhibition (p<0.001). P_2_Y and PI3K p110β inhibition alone had no effect on hypothermic ECC-induced CD11b expression on granulocytes.

## Discussion

**Figure 6:**
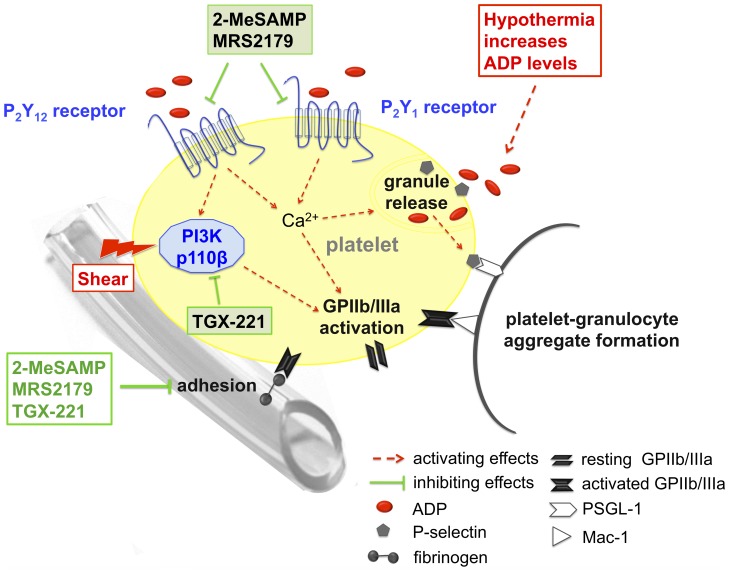
Overview of a pharmacological strategy for platelet protection during hypothermic ECC employing P_2_Y_12_ and P_2_Y_1_ receptor blockers as well as a PI3K p110β inhibitor.

In this study, we investigate the effects of ADP receptor blockade combined with PI3K p110β inhibition on platelets and granulocytes in an *ex vivo* model simulating hypothermic ECC. In general, our current findings confirm previously reported effects of hypothermic ECC on platelet activation, which lead to numerous responses in platelets like: platelet adhesion to the ECC surface, granule release associated with upregulation of P-selectin on platelets and PMPs, activation of GPIIb/IIIa and subsequent interaction of platelets and granulocytes as well as granulocyte activation (Figure 6) [1], [2], [12], [31], [32], [33], [34]. In our current study, GPIbα and GPIIb/IIIa expression levels as well as vWF binding to platelets were unaffected by hypothermic ECC. Hypothermic ECC did also not result in increased PMP numbers, but in an increase of P-selectin expression on PMPs and single platelets. This contributes to explain the increase in platelet-granulocyte aggregate formation and upregulation of Mac-1 expression on granulocytes as observed in our experiments, since P-selectin expressing platelets and PMPs can mediate binding and consecutive activation of platelets and leukocytes [35]. Furthermore, the observed loss of platelet counts after hypothermic ECC can be explained by the fact that aggregate formation as well as platelet adhesion to the ECC surface occurred during hypothermic ECC.

Based on these data and previous findings [3], [10], [36], a routine strategy for platelet protection during ECC and hypothermia is warranted and could be very beneficial for many patients in clinical settings where ECC and hypothermia are employed.

In patients with coronary disease platelet inhibition is routinely performed with ADP receptor P_2_Y_12_ blockers including the thienopyridines clopidogrel and prasugrel [37], [38]. We have recently reported that administration of cangrelor, a reversible non-thienopyridine P_2_Y_12_ receptor blocker with short plasma half-life (3–6 minutes) [39] effectively reduces platelet granule release, platelet-granulocyte binding, and platelet loss during hypothermic ECC [11]. Furthermore, according to its short half-life administration of cangrelor only during ECC results in rapid recovery of platelet function after ECC and is not associated with blood loss. Nevertheless, cangrelor had no inhibitory effects on platelet-ECC binding and no sustainable effect on GPIIb/IIIa activation [11]. In the setting of normothermic *ex vivo* ECC (37°C), platelet-ECC adhesion as well as platelet-platelet and platelet-granulocyte aggregation can be very effectively decreased with the PI3K p110β inhibitor TGX-221 [4]. In addition, further inhibition of ADP-mediated effects may be achieved by blocking not only P_2_Y_12_, but also P_2_Y_1_ on platelets. Therefore, the aim of our current study was to combine full platelet ADP receptor blockade with inhibition of shear-induced platelet activation to completely inhibit platelet activation during ECC and hypothermia.

At first, we employed a combination of experimental P_2_Y_12_ and P_2_Y_1_ blockers to protect platelets during hypothermic ECC. The P_2_Y_1_ receptor has been suggested as a crucial target for inhibition of ADP-mediated effects, since P_2_Y_1_ activation is necessary for full platelet aggregation [8]. Nevertheless, until today, no P_2_Y_1_ antagonist for clinical applications has been developed [40], [41]. We therefore used the experimental and reversible P_2_Y_1_ antagonist MRS2179, which has been shown to substantially inhibit ADP-induced platelet aggregation [42]. Furthermore, 2-MeSAMP, a specific inhibitor of the platelet P_2_Y_12_ receptor [43], [44], was used in the current study, because previous *ex vivo* experiments as well as our current *in vitro* experiments showed substantial and specific P_2_Y_12_ blockade to a similar extent then the effect observed with cangrelor [10], [11].

Our results show, that combined P_2_Y_12_ and P_2_Y_1_ blockade results in significant inhibition of P-selectin expression on platelets and PMPs as well as a reduction in platelet-granulocyte aggregates. Nevertheless, similar to results observed in a previous study [11], no effect of P_2_Y_12_ and P_2_Y_1_ blockade was observed for GPIIb/IIIa activation and platelet-ECC adhesion.

Jackson *et* *al*. have reported the platelet PI3K p110β isoform as important target for new antithrombotic agents, since it plays a major role in shear-induced activation of platelets [45]. In order to inhibit the platelet PI3K isoform p110β, TGX-221, an experimental compound with a reported 1.000-fold selectivity over the two other PI3K isoforms p110α and p110γ [45], was used. Only the combined administration of P_2_Y blockade and PI3K p110β inhibition prevented platelet-ECC adhesion during hypothermic ECC and provided a better protection of platelet counts and inhibition of GPIIb/IIIa activation then P_2_Y blockers or TGX-221 alone.

Overall, combined blockade of P_2_Y_12_, P_2_Y_1_ and PI3K p110β during hypothermic ECC in our current experiments resulted in an effective reduction of all investigated platelet activation markers. Furthermore, the effect of combined blockade of P_2_Y_12_, P_2_Y_1_ and PI3K p110β achieved inhibitory effects close to values measured at baseline before ECC and is therefore more profound then the sole inhibition of P_2_Y_12_, P_2_Y_1_ or PI3K p110β. This confirms that activation processes mediated via ADP and shear stress in combination are the major players for the induction of platelet activation during hypothermic ECC. Therefore, inhibition of the respective receptors and activation pathways may be very beneficial for patients undergoing hypothermic ECC. As a consequence the development of respective agents for use in clinical applications is highly desirable. Such innovative substances for administration during ECC and hypothermia should optimally have a short half-life. In this case platelet inhibition can be achieved only during the phase of ECC and hypothermia and platelet function is quickly restored after termination of infusion after ECC. We have recently demonstrated the functionality of this innovative pharmacological principle by employing cangrelor during hypothermic ECC *in vivo* [11].

In conclusion, our novel findings indicate that hypothermic ECC-induced platelet activation as well as subsequent events including aggregate formation and granulocyte activation are completely inhibited by combined P_2_Y_12_, P_2_Y_1_ and PI3K p110β blockade. This therapeutic strategy carries the potential to effectively protect platelets and to prevent life-threatening complications like thrombosis, bleeding and systemic inflammation in the setting of ECC and hypothermia. Hence, our findings warrant additional studies to further evaluate this new antiplatelet strategy and to develop innovative reversible antiplatelet agents for clinical use.

### Limitations and Outlook

The Chandler loop ECC model, which was used for the *ex vivo* ECC experiments in this study, is a well-established and routinely used model to analyze effects of ECC and hypothermia on platelets and other blood components. However, the obtained results need to be interpreted with caution, since the Chandler loop model differs in some points from the setting of ECC as employed in cardiac surgery. In the Chandler loop PVC tubings, which contain human whole blood, are circulated in a water bath at a designated temperature. Therefore, human whole blood is circulated exclusively and continuously over artificial surfaces, which causes activation of platelets and leukocytes. However, in the Chandler loop model heart-lung machine components like oxygenator and roller pumps, which are routinely used during cardiac surgery, are omitted and therefore shear stress and other activating effects might be decreased in comparison to what happens under clinical conditions. Furthermore, no priming volume is used in the Chandler loop model, which may result in augmented activating effects. Nevertheless, it has been shown in previous studies that platelet activation is induced during cardiac operations employing a heart-lung machine and hypothermia to a similar extent compared to what we found in our experiments [1], [36], [46], [47].

Our findings indicate that combined P_2_Y and PI3K inhibition has the potential to achieve complete platelet inhibition during hypothermic ECC and may therefore be very beneficial in the clinical situation. Therefore, our final aim is to evaluate this antiplatelet strategy in a carefully designed clinical study. However, previous studies employing different animal models revealed that there is a species-dependent variation regarding the effect of TGX-221 on bleeding [45], [48]. Therefore, before a clinical investigation may be initiated, the effect of our experimental strategy on the bleeding time needs to be carefully evaluated in human blood. Especially administration of short-acting drugs like cangrelor or drugs, which doǹt influence the bleeding time, will be the agents of choice for the *in vivo* setting and should be further evaluated in the future.

## Methods

### Blood Sampling

All blood sampling procedures were approved by the Research and Ethics Unit of the University of Tübingen, Germany (project number 270/2010BO1). Written informed consent was obtained from all subjects before blood sampling.

Blood from healthy donors was collected by venipuncture and anticoagulated with heparin [final concentration (fc): 3 I.U./ml]. All subjects were free of platelet-affecting drugs for at least 14 days.

### *In vitro* Evaluation of Different Concentrations of 2-MeSAMP and TGX-221

Heparinized human whole blood (n = 4) was treated for 5 minutes at 37°C with the following agents: PBS as control (Invitrogen GmbH, Karlsruhe, Germany), 2-MeSAMP (fc: 10 and 100 µM; dissolved in PBS; Sigma-Aldrich Corporation, St. Louis, USA), a combination of 2-MeSAMP and MRS2179 (fc: 100 µM each; dissolved in PBS; Tocris Biosciences, Bristol, UK), propylene glycol (PG; Carl Roth GmbH + Co. KG, Karlsruhe, Germany) as control or TGX-221 (fc: 0.5 or 2.2 µM; dissolved in PG; Merck Chemicals Ltd., Nottingham, UK). Afterwards whole blood was incubated with ADP (fc: 20 µM) and a phycoerythrin (PE)-labeled anti-P-selectin monoclonal antibody (mAb) (BD Biosciences, Heidelberg, Germany) as well as an fluorescein isothiocyanate (FITC)-labeled anti-GPIIb mAb (Beckman Coulter, Marseille, France) according to previously described methods for 20 minutes at 37°C [3]. Next, all samples were fixed using CellFix® (BD Biosciences) and flow cytometric analysis was performed within 6 hours.

### *Ex vivo* ECC Chandler Loop Model

In each experiment, baseline values were measured directly after blood sampling in 20 ml of heparinized blood. A well-established closed-loop ECC model (Chandler loop) [17] and PVC tubings (tubing length: 50 cm, inner diameter: 7,6 mm; wall: 2,3 mm) without additional coating (Jostra, Hirrlingen, Germany) were used to simulate ECC at 28°C. For each donor, five tubings were filled with 20 ml of fresh heparinized whole blood and treated with the following agents: the first and second sub-samples were treated with PBS and PG as controls, respectively. In the third sub-sample, both platelet ADP receptors were blocked (“P_2_Y block”) employing the experimental P_2_Y_12_ antagonist 2-MeSAMP (fc: 100 µM) and the experimental P_2_Y_1_ antagonist MRS2179 (fc: 100 µM). In the fourth sub-sample, PI3K p110β was inhibited by TGX-221 (fc: 2.2 µM). In the last sub-sample, a combination of P_2_Y blockade and PI3K p110β inhibition was employed. Afterwards, each tubing was closed into a circuit and circulated in a water bath (at 30 rpm) at 28°C for 30 minutes.

In this study, blood was anticoagulated with heparin, because heparin is routinely applied during clinical procedures employing ECC as anticoagulant to prevent activation of plasmatic coagulation during ECC. The degree of potential platelet activating effects of heparin on platelets has been analyzed in a previous study and found to be of minor extent in our *ex vivo* ECC model [3].

### Flow Cytometric Analyses of Blood Samples Before and After ECC

Analyses of human platelets and granulocytes were performed directly after blood sampling (“before circulation”) and after *ex vivo* ECC in all treatment groups. Expression of P-selectin, GPIbα as well as binding of the PAC-1 mAb against activated GPIIb/IIIa on platelets was measured according to previously described methods [3], [10].

Expression of the GPIIb/IIIa receptor on platelets as well as platelet von Willebrand factor (vWF) binding was evaluated in 25 µl of diluted whole blood (1∶50 dilution in modified Tyrodès buffer) using 5 µl of an anti-GPIIb-FITC (Beckman Coulter) and 5 µl of an anti-vWF-FITC mAb (1∶10 dilution; Abcam, Cambridge, UK), respectively. P-selectin and GPIbα expression as well as vWF binding were also analyzed on ADP-activated (fc: 20 µM) platelets before and after hypothermic ECC in the PBS group as positive controls.

Analysis of platelet microparticles (PMPs) was performed after flow cytometric calibration and setup using a forward scatter/sideward scatter dot plot and 1 and 3 µm-sized latex beads (Polysciences, Eppelheim, Germany). PMPs are defined as objects with a size of ≤1 µm. The number of PMPs was counted and P-selectin expression was analyzed as described previously [3]. Platelet-granulocyte aggregates were detected in 25 µl of whole blood, which was incubated for 20 minutes with 5 µl of an anti-GPIIb-FITC mAb and 5 µl of an anti-CD15-PE mAb. Afterwards, samples were treated with FACS Lysing Solution (BD Biosciences) to lyse erythrocytes, centrifuged at 200 g for 5 minutes and washed with PBS.

CellFix® (BD Biosciences) was used for sample fixation. Flow cytometry was performed within 6 hours using a FACScan® cytometer (BD Biosciences) according to standard procedures [3], [18].

For evaluation of granulocyte activation, 90 µl of whole blood was incubated with 10 µl of an anti-CD11b-PE mAb (Beckman Coulter) for 20 minutes at 37°C, treated with FACS Lysing Solution, centrifuged at 200 g for 5 minutes and afterwards washed with PBS. All samples were fixed using CellFix®. Within 6 hours after antibody staining, granulocytes were identified according to their size and granularity and analyzed using flow cytometry.

For all samples, suitable isotype antibodies were used to adjust fluorescence amplification settings. A total of 10.000 events were acquired in each sample. If not otherwise indicated, geometric mean fluorescence intensities were used for analyses of flow cytometric data.

### Detection of Platelet Adhesion to the ECC Surface

Platelet adhesion to the ECC surface was detected using a specially designed enzyme-linked immunosorbent assay (ELISA) method as previously described [4]. Briefly, ECC tubings were washed and blocked after circulation and surface-bound GPIIb was detected employing a primary anti-GPIIb antibody (Sigma, Deisenhofen, Germany) and an alkaline phosphatase conjugated secondary antibody (Immunotech/Coulter, Marseille, France). The chromogenic reaction was stopped by addition of NaOH. Light absorbance was determined with an ELISA reader MR 5000 (Dynatech, Denkendorf, Germany) at 405 nm.

### Analysis of Platelet Counts

Before and after circulation, human whole blood was anticoagulated with EDTA (EDTA-Monovette®, Sarstedt, Nümbrecht, Germany) for platelet count analysis using an ABX Micros 60 blood analyzer (Axon Lab AG, Baden-Dättwil, Switzerland).

### Statistical Analysis

Data are depicted as means with standard deviation (SD). In order to test data sets for normality, the Kolmogorov-Smirnov test was performed. Normally distributed data were then analyzed using repeated measures (RM) ANOVA with Bonferroni’s multiple comparison test to analyze differences between groups, while not normally distributed data were analyzed using a non-parametrical test (Friedman test with Dunǹs multiple comparison test). All analyses were performed using the statistical software program GraphPad Prism (version 5, GraphPad Software, La Jolla, USA). Statistical significance was defined as p<0.05.
